# Cold Exposure Promotes Atherosclerotic Plaque Growth and Instability via UCP1-Dependent Lipolysis

**DOI:** 10.1016/j.cmet.2013.06.003

**Published:** 2013-07-02

**Authors:** Mei Dong, Xiaoyan Yang, Sharon Lim, Ziquan Cao, Jennifer Honek, Huixia Lu, Cheng Zhang, Takahiro Seki, Kayoko Hosaka, Eric Wahlberg, Jianmin Yang, Lei Zhang, Toste Länne, Baocun Sun, Xuri Li, Yizhi Liu, Yun Zhang, Yihai Cao

**Affiliations:** 1 The Key Laboratory of Cardiovascular Remodeling and Function Research, Chinese Ministry of Education and Chinese Ministry of Health, Shandong University Qilu Hospital, Jinan, Shandong 250012, People’s Republic of China; 2 Department of Microbiology, Tumor and Cell Biology, Karolinska Institute, 171 77 Stockholm, Sweden; 3 Department of Medicine and Health Sciences, Linköping University, 581 83 Linköping, Sweden; 4 Department of Pathology, Tianjin Medical University, Tianjin 300070, People’s Republic of China; 5 State Key Laboratory of Ophthalmology, Zhongshan Ophthalmic Center, Sun Yat-Sen University, Guangzhou 510060, People’s Republic of China

## Abstract

Molecular mechanisms underlying the cold-associated high cardiovascular risk remain unknown. Here, we show that the cold-triggered food-intake-independent lipolysis significantly increased plasma levels of small low-density lipoprotein (LDL) remnants, leading to accelerated development of atherosclerotic lesions in mice. In two genetic mouse knockout models (apolipoprotein E^−/−^ [ApoE^−/−^] and LDL receptor^−/−^ [Ldlr^−/−^] mice), persistent cold exposure stimulated atherosclerotic plaque growth by increasing lipid deposition. Furthermore, marked increase of inflammatory cells and plaque-associated microvessels were detected in the cold-acclimated ApoE^−/−^ and Ldlr^−/−^ mice, leading to plaque instability. Deletion of uncoupling protein 1 (UCP1), a key mitochondrial protein involved in thermogenesis in brown adipose tissue (BAT), in the ApoE^−/−^ strain completely protected mice from the cold-induced atherosclerotic lesions. Cold acclimation markedly reduced plasma levels of adiponectin, and systemic delivery of adiponectin protected ApoE^−/−^ mice from plaque development. These findings provide mechanistic insights on low-temperature-associated cardiovascular risks.

## Highlights

• Cold activates lipolysis and increases blood levels of VLDL and LDL remnants
• Cold-induced hypercholesterolemia promotes plaque growth and instability
• Deletion of *Ucp1* attenuates cold-induced lipolysis, plaque growth, and instability
• Cold acclimation reduces plasma adiponectin levels

## Graphical Abstract

**Figure:**
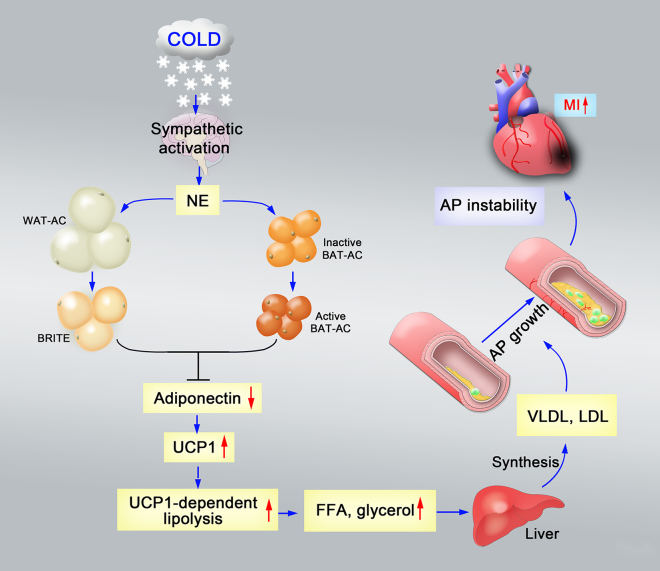

## Introduction

Atherosclerosis-associated cardiovascular disease (CVD) remains the leading cause of mortality in the Western society and most other parts of the world (Libby et al., 2011; Maseri et al., 2011). Recent studies show that colder ambient temperature is associated with an increased incidence of myocardial infarction (MI) and other atherosclerosis-related vascular disorders such as stroke (Bhaskaran et al., 2009; Palmer, 2010). Abundant evidence from epidemiological studies has linked cardiovascular disorders to the cold seasons or low ambient temperature (The Eurowinter Group, 1997; Barnett, 2007; Basu et al., 2005; Braga et al., 2001; Morabito et al., 2005; Wolf et al., 2009). Despite this long-known linkage between cold and high CVD risk, mechanisms underlying cold-induced CVD remain poorly understood, although snow removal during the winter season has been claimed to be associated with increased CVD incidence (Franklin et al., 2001; Janardhanan et al., 2010).

In rodents, cold exposure can sufficiently activate brown adipose tissue (BAT), leading to increased levels of nonshivering thermogenesis via activation of the sympathetic system (Lowell and Spiegelman, 2000; Nedergaard et al., 2001; Rosen and Spiegelman, 2006; Shabalina et al., 2006; Xue et al., 2009). Additionally, low ambient temperature can sufficiently stimulate phenotypic and functional conversion from white adipose tissue (WAT) into brown-like adipose tissue (BRITE) in an uncoupling protein 1 (UCP1)-dependent manner (Nedergaard et al., 2001; Petrovic et al., 2010; Xue et al., 2009). Interestingly, the cold-induced transition from WAT into BRITE is also associated with an increased density of blood vessels that provide oxygen as a fuel to cope with the high rate of metabolism (Cao, 2010; Xue et al., 2009). A substantial amount of BAT exists in adult humans, and cold augments BAT activation (Celi, 2009; Cypess et al., 2009; Enerbäck, 2009; Timmons and Pedersen, 2009; van Marken Lichtenbelt et al., 2009; Virtanen et al., 2009), which may have a paradoxical impact on health. In a healthy adult, BAT activation may lead to a lean and healthier phenotype. Conversely, cold-triggered high metabolic rates may alter plasma lipid profiles, leading to accelerated development and progression of metabolically related disorders, such as atherosclerosis and diabetes, if an individual already suffers from this type of disorder. The latter possibility has not been investigated. Atherosclerotic plaques typically occur in the aortic, coronary, carotid, and cerebral arteries (Libby et al., 2011) and thus are closely associated with high incidences of CVD and stroke (Eaton, 2005). Deletion of apolipoprotein E (*ApoE*) and low-density lipoprotein receptor (*Ldlr*) in mice leads to development of atherosclerotic lesions in the aorta and its branches, due to increased plasma levels of LDL cholesterol (Oka et al., 2001; Powell-Braxton et al., 1998).

In this study, we investigated the impact of cold-induced BAT activation-associated lipolysis on atherosclerotic plaque formation and stability in both ApoE^−/−^ and Ldlr^−/−^ mice. We report our findings that cold exposure significantly accelerated the atherosclerotic plaque growth and instability in both genetic models by the mechanism of accelerated UCP1-dependent lipolysis. Cold-associated reduction of plasma adiponectin levels is significantly involved in the accelerated development of atherosclerotic plaques. Our work sheds light on the mechanisms that underlie cold-induced cardiovascular risks that may pave an avenue for the treatment and prevention of atherosclerosis-associated CVD.

## Results

### Cold Exposure Activates BAT and Induces BRITE

**Figure 1:**
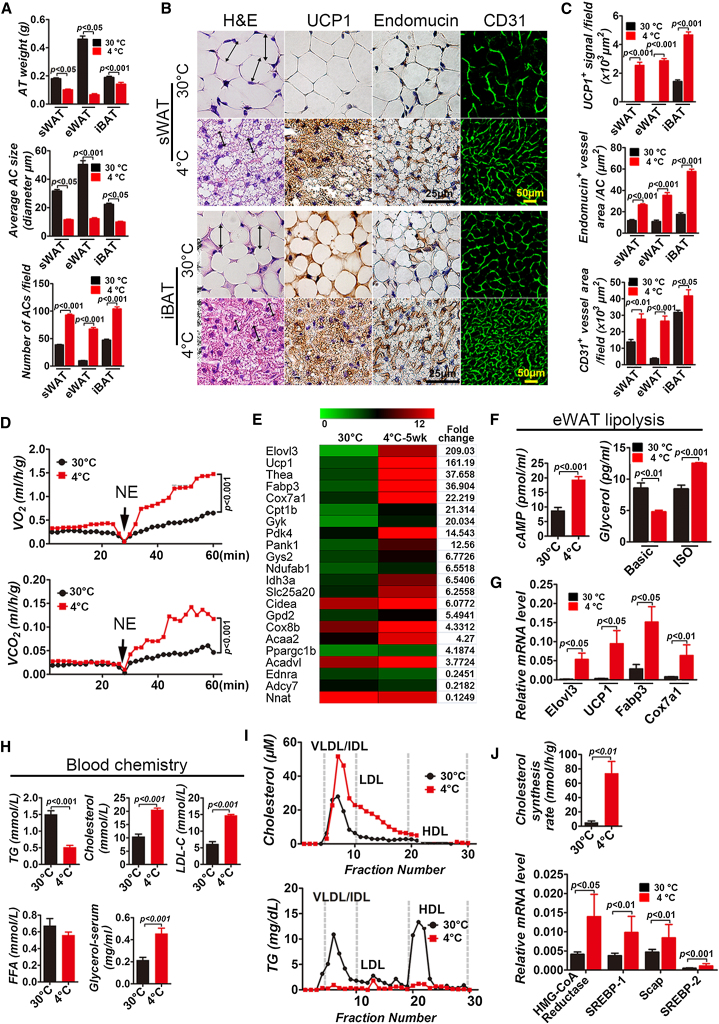
Cold-Induced WAT-BRITE Conversion, BAT Activation, Blood Lipid Profile, Lipolysis, and Cholesterol Synthesis of ApoE^−/−^ Mice (A) Tissue weights of sWAT, eWAT, and iBAT depots from ApoE^−/−^ mice exposed to 4°C or 30°C for 8 weeks (n = 20/group). Average diameters and average numbers of adipocytes of sWAT, eWAT, and iBAT exposed to 4°C or 30°C for 8 weeks (10 random fields/group × 5 mice). (B) Tissue sections of sWAT and iBAT exposed to 4°C or 30°C for 8 weeks were stained with H&E, mitochondrial UCP1, endomucin, and CD31. (C) Quantification of UCP1^+^ signals, endomucin^+^ microvessel density per adipocyte, and CD31^+^ microvessel density of sWAT, eWAT and iBAT exposed to 4°C or 30°C for 8 weeks (10 random fields/group × 5 mice). (D) Metabolic rate of O_2_ consumption and CO_2_ production in response to NE (n = 7/group). (E) Expression profiles of lipolysis-related genes in iWAT exposed to 4°C or 30°C for 5 weeks (3 repeats/group). (F) Measurement of cAMP and glycerol levels in eWAT exposed to 4°C or 30°C for 8 weeks (n = 5/group). (G) qPCR analysis of expression levels of lipolysis-related genes in iWAT exposed to 4°C or 30°C for 8 weeks (n = 6/group). (H) Blood chemistry analysis of TG, cholesterol, LDL cholesterol (LDL-C), FFA, and glycerol (n = 12/group). (I) FPLC analysis of plasma cholesterol and TG in groups exposed to 4°C or 30°C for 8 weeks (n = 6/group). (J) In vivo analysis of the hepatic cholesterol synthesis rate in groups exposed to 4°C or 30°C for 8 weeks (n = 6/group). qPCR analysis of expression levels of cholesterol synthesis-related genes from liver tissues of groups exposed to 4°C or 30°C for 8 weeks (n = 6/group). AT, adipose tissue; AC, adipocyte; NE, norepinephrine.

To study the effect of low temperature on alterations of both WAT and BAT in ApoE^−/−^ mice, 8-week-old adult animals that were fed with a high-fat diet (HFD) for 4 weeks were divided into 2 groups (n = 20/group). One group of mice was transferred to 30°C, the thermoneutral temperature; under this condition, the metabolic rate remained at the basal level. The other group of mice was adapted to 18°C for 1 week prior to exposure to 4°C. Mice in both groups were pair-fed with regular chow for subsequent studies. At the end of week 8 after cold exposure, total weights of subcutaneous WAT (sWAT) and epididymal WAT (eWAT) were significantly reduced (Figure 1A). Histological analysis of sWAT showed that the average size of the adipocytes was markedly smaller in the cold-exposed group as compared with those exposed to the thermoneutral temperature (Figures 1A and 1B). Due to a smaller adipocyte size, the number of adipocytes per field was relatively increased (Figure 1A).

Cellular contents of 4°C sWAT were markedly increased and contained a high density of hematoxylin and eosin (H&E)-stained structures (Figure 1B). Immunohistochemical analysis demonstrated that high expression levels of UCP1, a specific mitochondrial protein for BAT, were present in 4°C sWAT, whereas UCP1 levels were completely undetectable in 30°C sWAT (Figures 1B and 1C). Kinetic studies demonstrated that these alterations in WATs were readily detected after 4-week exposure to cold (Figure S1 available online). These findings demonstrate that exposure of ApoE^−/−^ mice to cold led to conversion of WAT in various depots into BRITE. Cold exposure also augmented the activation of interscapular BAT (iBAT) by increasing UCP1 expression (Figures 1B and 1C). In addition to alterations of adipocytes, the microvessel density in 4°C WATs and 4°C iBAT was significantly increased (Figures 1B and 1C), validating the fact that cold exposure could switch to an angiogenic phenotype in the adipose tissue (Xue et al., 2009).

### Activation of Lipolysis and Metabolism by Cold Exposure

Nonshivering thermogenesis-related metabolism can be detected by measuring the metabolic rate in response to norepinephrine (NE) (Xue et al., 2009). As expected, exposure of ApoE^−/−^ mice to cold for 8 or 4 weeks led to marked elevation of the metabolic rate as measured by O_2_ consumption and CO_2_ production (Figures 1D and S1D). These findings are consistent with the decrease of body weight and body mass index (BMI) due to a high metabolic rate during cold acclimation (Figure S1C). The cold-triggered high metabolic rate and lipolysis are likely to alter blood lipid profiles. Indeed, the level of plasma triglyceride (TG) was significantly decreased (Figure 1H), further validating the cold-induced body weight loss. Conversely, levels of cholesterol, LDL cholesterol, and glycerol were increased by 2- to 3-fold, whereas the level of free fatty acid (FFA) remained unchanged (Figure 1H). Fast protein liquid chromatography (FPLC) analysis of plasma cholesterol under cold exposure showed that the proportion of very low-density lipoprotein (VLDL) was particularly increased (Figure 1I). Similarly, intermediate-density lipoprotein (IDL) and the proportion of LDL were also markedly increased by cold exposure. Conversely, plasma levels of the high-density lipoprotein (HDL) cholesterol component remained virtually unchanged (Figure 1I). We next studied cholesterol synthesis by intraperitoneal injection with [^3^H]water. Intriguingly, the hepatic cholesterol synthesis rate was markedly increased in cold-exposed ApoE^−/−^ mice (Figure 1J). Consistent with the increased level of cholesterol synthesis, hydroxymethylglutaryl-coenzyme A reductase (HMG-CoA reductase) and transcriptional factors essential for cholesterol synthesis, including sterol regulatory element-binding proteins (SREBPs) transcription factor and its partner protein SREBP cleavage-activating protein (SCAP), were also markedly increased (Figure 1J). Inversely, plasma levels of TG were markedly decreased in cold-exposed ApoE^−/−^ mice relative to those of the thermoneutral temperature-exposed group (Figure 1H). Similar results were also obtained from mice exposed to cold for 4 weeks (Figure S1H).

To exclude the effect of HFD in cold-induced hyperlipidemia, we performed experiments in which mice were fed with regular diet throughout the entire experiment. As expected, similar cold-induced hypercholesterolemia, plaque growth, and instability were also seen in these regular chow-fed ApoE^−/−^ mice (Figures S3B–S3D). These results show that cold-triggered metabolic changes in the adipose tissue alter the blood lipid profiles by accumulating atherosclerosis-prone lipids. Since the sympathetic system is involved in activation of BAT- and BRITE-associated lipolysis (Xue et al., 2009), which subsequently regulates hepatic cholesterol synthesis, we treated ApoE^−/−^ mice with a known β3-adrenergic receptor-selective blocker, SR-59230A. Interestingly, SR-59230A significantly inhibited the cold-induced hypercholesterolemia, plaque development, and instability (see below for description of measures of stability; Figures S3F–S3H).

We also analyzed blood lipid profiles in cold-exposed healthy mice. Similar to ApoE^−/−^ mice, prolonged exposure to cold significantly decreased body weight and BMI in genetically nonmanipulated healthy mice (Figure S4A). Plasma levels of TG were significantly decreased (Figure S4B). However, plasma levels of total cholesterol, LDL cholesterol, and HDL cholesterol were all significantly decreased (Figure S4B). These findings opposed the high cholesterol levels seen in ApoE^−/−^ mice. One possible explanation of these discrepant findings between healthy and ApoE^−/−^ mice could be the high rate of cholesterol clearance in healthy mice. In support of this notion, hepatic *Ldlr* and *ApoE* levels were markedly increased in cold-exposed healthy mice (Figure S4C). In addition to the pair-fed protocol, ad libitum fed ApoE^−/−^ mice also exhibited high plasma levels of total cholesterol and LDL cholesterols in response to cold exposure (Figure S4E). Consistent with cold-induced hypercholesterolemia, accelerated plaque growth and instability were also observed in these ad libitum fed mice (Figures S4F and S4G). We have also measured the plasma levels of corticosterone. There was no statistical difference between the 4°C and 30°C groups after 4 weeks of exposure. However, a statistical difference has been observed after prolonged exposure (8 weeks), suggesting that mice experienced chronic stress (Figure S1G).

### Elevated Expression Levels of Lipolysis-Associated Gene Products

To study alteration of expression levels of metabolically associated genes, a genome-wide microarray analysis was performed in four groups (4°C for 1 week, 4°C for 5 weeks, 30°C for 1 week, and 30°C for 5 weeks) using the inguinal WAT (iWAT), which exhibited an obvious BRITE phenotype. Gene cluster and principle component analyses demonstrated that gene expression patterns were highly reproducible. As expected, a number of lipolysis-related genes, including *fabp3*, *ucp1*, *elovl3*, *cpt1b*, *thea*, *cox7a1*, *gyk*, *pank1*, *pdk4*, *cox8b*, *idh3a*, *gys2*, *gpd2*, *acdav1*, *ndufab*, *acaa2*, and *pparg1b*, were highly expressed in 4°C for 1 week or 4°C for 5 week iWAT (Figures 1E and S1B). Noticeably, many of these cold, highly-induced lipolysis-associated genes are crucial enzymes for oxidative and lipid degradation. Consistent with gene profile alterations, high expression levels of several key lipolysis genes were further validated by quantitative real-time PCR (qPCR) analysis (Figures 1F and S1E). To further define the molecular players that are involved in lipolysis in the adipose tissue, expression levels of cyclic AMP (cAMP), an essential signaling component of lipolysis in the adipose tissue, were analyzed in 4°C eWAT and 30°C eWAT. Interestingly, the cAMP level in the 4°C eWAT group was significantly increased relative to the 30°C eWAT group (Figures 1F and S1F). Furthermore, adipocytes from 4°C eWAT and 30°C eWAT were isolated and incubated in the presence or absence of isoproterenol (ISO). As expected, stimulation of ISO-treated 4°C eWAT adipocytes significantly increased the release of glycerol, indicating that the high rate of lipolysis occurred in these cells.

### Cold Exposure Stimulates Atherosclerotic Plaque Growth in ApoE^−/−^ Mice

**Figure 2:**
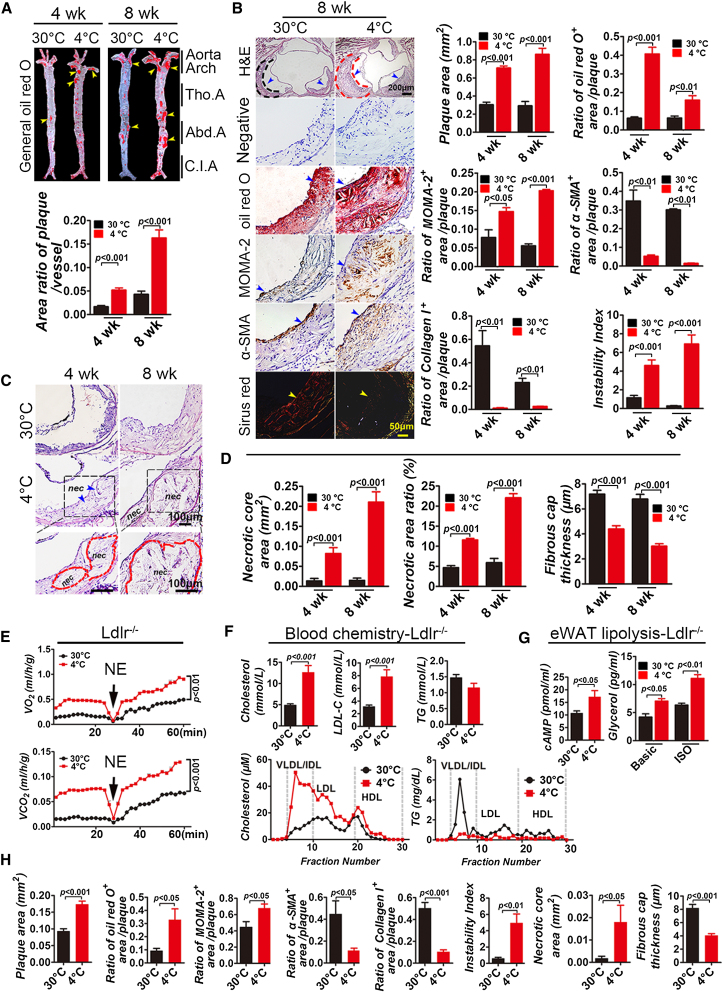
Cold-Induced Atherosclerotic Plaque Growth and Instability in ApoE^−/−^ Mice and Ldlr^−/−^ Mice (A) Gross examination and quantification of the general oil red O-stained aorta stem from ApoE^−/−^ mice exposed to 4°C and 30°C. (n = 10/group). (B) Cross-section histological analysis and quantification of aorta roots exposed to 4°C and 30°C for 8 weeks by staining with H&E, oil red O, MOMA-2, α-SMA, or Sirius red. Nonimmune IgG was used as negative control. Dashed lines encircle some parts of atherosclerotic plaques, and arrowheads in different panels point to positive signals (n = 10/group). (C) Dashed lines encircle the necrotic core area. nec, necrotic core. (D) Quantification of average necrotic core areas, the ratio of the necrotic core area versus the aortic root plaque area, and the fibrous cap thickness in groups exposed to 4°C and 30°C (n = 10/group). (E) Metabolic rate of O_2_ consumption and CO_2_ production in response to NE in Ldlr^−/−^ mice (n = 6–7/group). (F) Blood chemistry analysis of TG, cholesterol, and LDL levels (n = 8/group). FPLC analysis of plasma cholesterol and TG in pair-fed Ldlr^−/−^ mice exposed to 4°C and 30°C (n = 5/group). (G) Measurement of cAMP and glycerol release in eWAT of Ldlr^−/−^ mice exposed to 4°C and 30°C for 8 weeks (n = 4–5/group). (H) Quantification of plaque area and histological analysis of atherosclerotic plaques of aortic root in Ldlr^−/−^ mice exposed to 4°C and 30°C (n = 10/group). Tho.A, thoracic artery; Abd.A, abdominal artery; C.I.A, common iliac artery.

Knowing that cold acclimation increased plasma levels of VLDL and small LDL remnants in ApoE^−/−^ mice, we next investigated the consequence of alteration of lipid profiles in affecting atherosclerotic plaque growth. Surprisingly, gross examination by general oil red O staining revealed that arteries, including the aorta arch, thoracic artery, abdominal artery, and common iliac artery, contained widespread atherosclerotic plaques throughout the artery stem after 4- and 8-week exposure to cold (Figure 2A). The average size of plaque areas was substantially increased after prolonged 8-week exposure to cold, though the plaques in the 30°C control group showed a tendency to grow at a slow rate (Figures 2A and 2B). The existence of large atherosclerotic plaques in the 4°C groups was further validated by oil red O staining (Figure 2B). Prolonged exposure of ApoE^−/−^ mice to cold for 8 weeks led to markedly accelerated atherosclerotic plaque growth, whereas the average plaque size in the 30°C group remained unchanged (Figures 2A and 2B). These findings demonstrate that cold significantly augments atherosclerotic plaque growth in main arteries of ApoE^−/−^ mice.

### Cold Acclimation Augments Atherosclerotic Plaque Instability in ApoE^−/−^ Mice

Immunohistochemical analysis showed that α-smooth muscle actin (α-SMA) positive structures and Sirius red^+^ collagen I structures in atherosclerotic plaques were significantly decreased after 4- and 8-week-long cold acclimation, while oil red O^+^ lipid area and MOMA-2^+^ area were significantly increased. (Figure 2B). In light of these changes, the plaque instability indexes in 4 week and 8 week 4°C groups were increased in several magnitudes relative to the 30°C control groups (Figure 2B). The plaque instability index was calculated according to the following formula: (oil red O^+^ area plus MOMA-2^+^ area) / (α-SMA^+^ area plus collagen I^+^ area). Atherosclerotic plaques in the group exposed to 4°C contained large necrotic core areas, which were progressively enlarged during prolonged exposure to cold (Figures 2C and 2D). Further, the thickness of the fibrous cap in plaques exposed to 4°C was significantly decreased relative to those of the group exposed to 30°C (Figure 2D). Both increases of necrotic areas and decreases of the fibrous cap thickness indicate that cold exposure increases plaque instability and potential plaque disruption. Notably, other plaque instability factors, including microvessels, inflammatory cells, and inflammatory cytokines, were all markedly increased in plaques exposed to 4°C (Figures S2D–S2G).

### Cold Acclimation Induces Atherosclerotic Plaque Growth and Instability in Ldlr^−/−^ Mice

To study if the cold-augmented atherosclerotic plaque growth and instability generally occurred in other mouse models and was not only restricted to ApoE^−/−^ mice, we used the Ldlr^−/−^ mouse strain (Powell-Braxton et al., 1998) to validate our findings. Similar to ApoE^−/−^ mice, nearly identical data were reproduced in the Ldlr^−/−^ mouse strain, including cold-induced WAT-to-BRITE transition, activation of BAT, adipocyte size changes and UCP1 upregulation, lipolysis, body weight and BMI decreases, cholesterol synthesis alterations of blood lipid profiles, plaque growth, and instability index (Figures 2E–2H and S2A–S2C). Taken together, these findings demonstrate that cold acclimation induces atherosclerotic plaque growth and instability in Ldlr^−/−^ mice; thus, our findings may be reasonably generalized to other mouse strains.

### Deletion of UCP1 Impairs Cold-Induced Lipolysis

**Figure 3:**
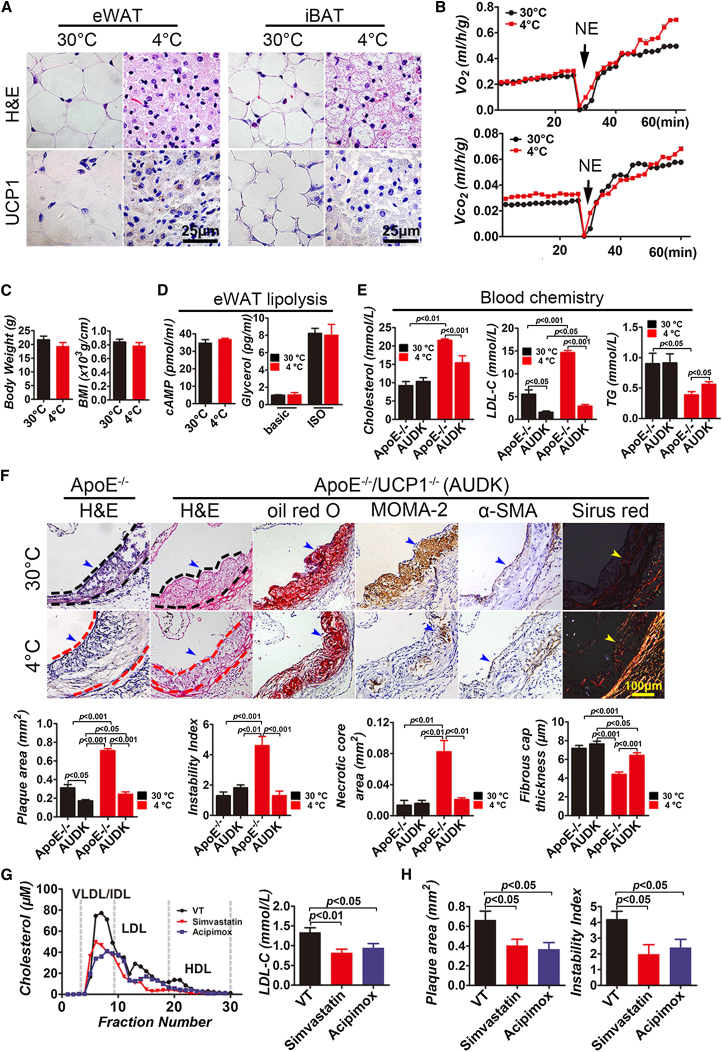
Plasma Cholesterol Level and Atherosclerotic Plaque in Simvastatin- and Acipimox-Treated ApoE^−/−^ Mice; Cold-Induced Alterations of BAT Activation, BMI, Metabolism, Blood Lipid Profile, Atherosclerotic Plaque Growth, and Stability in ApoE^−/−^/UCP1^−/−^ Mice (A) Histology and lack of UCP1 expression in eWAT and iBAT exposed to 4°C and 30°C for 4 weeks. (B) Metabolic rate of O_2_ consumption and CO_2_ production in response to NE (n = 5–6/group). No statistical significance between the two groups at any time points. (C) Body weight and BMI of pair-fed ApoE^−/−^/UCP1^−/−^ mice exposed to 4°C and 30°C (n = 6–7/group), showing no statistical significance between the two groups. (D) Measurement of cAMP and glycerol levels released from eWAT of ApoE^−/−^/UCP1^−/−^ mice exposed to 4°C and 30°C for 4 weeks (n = 4/group). No statistical significance between the two groups. (E) Blood chemistry analysis of cholesterol, LDL cholesterol, and TG levels of ApoE^−/−^/UCP1^−/−^ or ApoE^−/−^ mice exposed to 4°C and 30°C (n = 6/group). (F) Histological analysis of aorta roots of ApoE^−/−^/UCP1^−/−^ mice exposed to 4°C and 30°C for 4 weeks by staining with H&E, oil red O, MOMA-2, α-SMA, or Sirius red. H&E staining of the aorta roots of ApoE^−/−^ mice exposed to 4°C and 30°C for 4 weeks was used as a control. Dashed lines encircle some parts of atherosclerotic plaques, and arrowheads in different panels point to positive signals (n = 6–7/group). Quantification of plaque area, instability index, necrotic core areas, and fibrous cap thickness in ApoE^−/−^/UCP1^−/−^ and ApoE^−/−^ mice (n = 6–7/group). (G) FPLC analysis of plasma cholesterol in vehicle-, simvastatin- and acipimox-treated apoE^−/−^ mice (n = 5/group). (H) Quantification of plaque areas and instability index of vehicle-, statin-, and acipimox-treated groups (n = 10/group). VT, vehicle-treated; NE, norepinephrine; AUDK, ApoE^−/−^/UCP1^−/−^.

Since cold-induced nonshivering thermogenesis requires activation of UCP1 (Nedergaard et al., 2001), we generated double knockout mice that lacked ApoE and UCP1 (ApoE^−/−^/UCP1^−/−^). Immunohistological analysis confirmed that UCP1 expression in 4°C WAT and BAT was completely ablated in ApoE^−/−^/UCP1^−/−^ mice (Figure 3A). Cold-induced, nonshivering thermogenesis-related metabolism, body weight and BMI losses, and lipolysis were completely blocked in ApoE^−/−^/UCP1^−/−^ mice (Figures 3B–3D), which also suffered from shivering behavior. After 4 week of exposure to 4°C, blood cholesterol in ApoE^−/−^/UCP1^−/−^ mice was significantly decreased relative to that in ApoE^−/−^ mice, and a greater than 5-fold decrease of LDL cholesterol was detected (Figure 3E). Notably, deletion of the *Ucp1* gene also significantly reduced the basal level of LDL cholesterol in ApoE^−/−^/UCP1^−/−^ mice exposed to 30°C for 4 weeks, indicating that UCP1 mediates BAT and muscle metabolisms under the thermoneutral condition (Figure 3E). Consistently, deletion of UCP1 led to a slight, but significant, increase of blood TG in 4 week 4°C ApoE^−/−^/UCP1^−/−^ mice, demonstrating the impaired basal level of lipolysis (Figure 3E). These findings provide compelling evidence that deletion of UCP1 completely abolishes the cold-induced lipolysis in the adipose tissue.

### The Role of UCP1 in Atherosclerotic Growth

Cold-induced atherosclerotic plaque growth was inhibited in ApoE^−/−^/UCP1^−/−^ mice relative to ApoE^−/−^ mice (Figure 3F). The total amount of collagen I components was markedly increased in atherosclerotic plaques of 4°C ApoE^−/−^/UCP1^−/−^ mice relative to 30°C ApoE^−/−^/UCP1^−/−^ mice. In a sharp contrast to ApoE^−/−^ mice, deletion of UCP1 in 4 week 4°C ApoE^−/−^/UCP1^−/−^ mice led to an approximately 4-fold increase of atherosclerotic plaque stability as compared with ApoE^−/−^ mice exposed to 4°C for 4 weeks (Figure 3F). The plaque fibrous cap thickness in 4 week 4°C ApoE^−/−^/UCP1^−/−^ mice was considerably increased relative to 4 week 4°C ApoE^−/−^/UCP1^+/+^ mice (Figure 3F). Deletion of the Ucp1 gene in ApoE^−/−^ mice resulted in a marked decrease of cold-induced necrotic core area compared with that in 4°C ApoE^−/−^/UCP1^+/+^ mice.

### Inhibition of Lipolysis and Cholesterol Synthesis Attenuates Plaque Growth

To further validate our findings and strengthen our conclusions, we studied the impact of lipolysis and cholesterol synthesis on blood LDL levels and atherosclerotic plaque development. As expected, inhibition of lipolysis by a known inhibitor, acipimox (Guo et al., 2009), and inhibition of cholesterol synthesis by simvastatin (Meng et al., 2012) significantly attenuated cold-induced hyperlipidemia, atherosclerotic plaque growth, and instability (Figures 3G, 3H, and S3A). Consistent with this finding, blood levels of LDL cholesterol, but not HDL cholesterol, were significantly reduced by both drugs. Interestingly, treatment of cold-exposed ApoE^−/−^ mice with acipimox and simvastatin resulted in a similar antiatherosclerotic effect, suggesting that lipolysis and cholesterol synthesis are cohesively coupling processes that contribute to cold-induced atherosclerosis. These findings further validate our general conclusion that cold-induced lipolysis is the causative mechanism of atherosclerotic plaque development.

### Essential Role of Adiponectin in Mediating Cold-Induced Atherosclerosis

**Figure 4:**
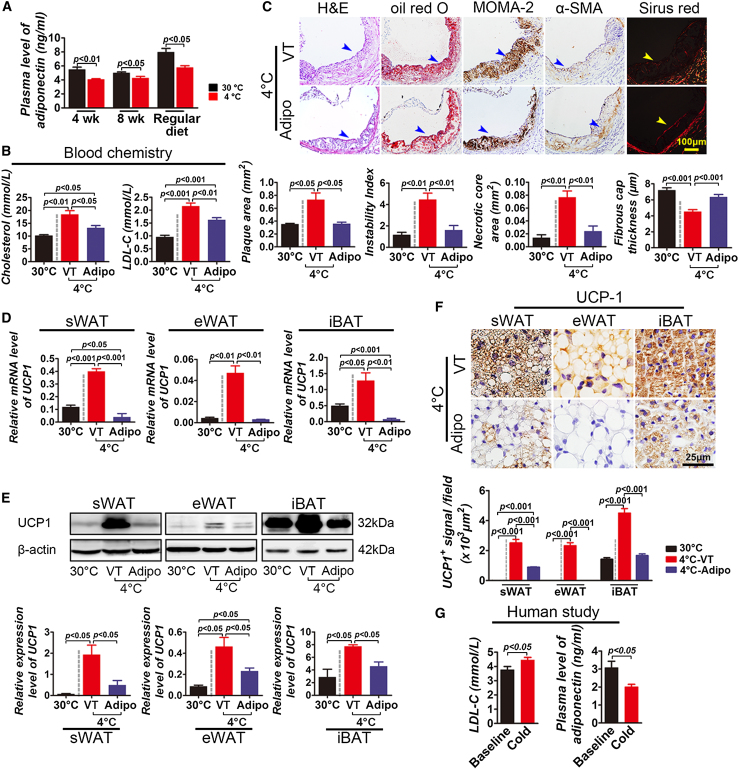
Adiponectin Treatment and Clinical Relevance (A) Plasma adiponectin levels of ApoE^−/−^ mice exposed to 4°C and 30°C (n = 10/group) that were fed HFD or regular diet for 4 and 8 weeks. (B) Plasma cholesterol and LDL cholesterol levels of adiponectin- or vehicle-treated ApoE^−/−^ mice exposed to 4°C or 30°C (n = 10/group). (C) Aorta root histological analysis of adiponectin- or vehicle-treated ApoE^−/−^ mice exposed to 4°C or 30°C (n = 10/group) stained with H&E, oil red O, MOMA-2, α-SMA, or Sirius red. Quantification of plaque areas, instability index, necrotic core areas, and fibrous cap thickness of adiponectin- or vehicle-treated ApoE^−/−^ mice exposed to 4°C or 30°C (n = 10/group). (D) Relative mRNA expression levels of UCP1 in sWAT, eWAT, and iBAT from adiponectin- or vehicle-treated ApoE^−/−^ mice exposed to 4°C or 30°C (n = 5/group). (E) Western blot analysis of UCP1 protein expression levels of sWAT, eWAT, and iBAT from adiponectin- or vehicle-treated ApoE^−/−^ mice exposed to 4°C or 30°C (n = 5/group). Quantification of protein levels of UCP1 in sWAT, eWAT, and iBAT from adiponectin- or vehicle-treated ApoE^−/−^ mice exposed to 4°C or 30°C (n = 5/group). (F) Histological analysis and quantification of UCP1 positive signals of sWAT, eWAT, and iBAT from adiponectin- or vehicle-treated ApoE^−/−^ mice exposed to 4°C or 30°C (n = 5/group). (G) Human plasma levels of LDL cholesterol and adiponectin after 2 days (2 hr/day) of cold exposure (n = 5). VT, vehicle-treated; Adipo, adiponectin-treated group.

To further elucidate molecular mechanisms that underlie cold-induced atherosclerotic plaque growth and instability, we analyzed adipokine levels in the plasma. Among the analyzed adipokines, plasma adiponectin levels were consistently decreased after 4 and 8 weeks of cold exposure in ApoE^−/−^ mice (Figure 4A). A similar level of plasma adiponectin reduction was also found in non-HFD-fed ApoE^−/−^ mice. A decreased level of adiponectin suggests that this adipokine might participate in regulation of lipid metabolism. We therefore investigated the role of adiponectin in modulation of lipolysis. Surprisingly, systemic delivery of adiponectin to cold-exposed ApoE^−/−^ mice substantially decreased levels of the total cholesterol and LDL cholesterol in the plasma (Figure 4B). Consistent with reduction of hypercholesterolemia, systemic administration of adiponectin inhibited plaque development to levels similar to that of plaques exposed to 30°C (Figure 4C). Similarly, adiponectin virtually completely ablated the effect of the cold-induced plaque instability (Figure 4C).

Since UCP1 is essentially required for cold-induced atherosclerotic plaque development in ApoE^−/−^ mice, we investigated the relation between adiponectin and UCP1 expression. Interestingly, systemic delivery of adiponectin almost completely inhibited UCP1 messenger RNA (mRNA) expression in iBAT, as detected by qPCR (Figure 4D). Consistent with decreases in mRNA levels, UCP1 protein levels in iBAT were also markedly decreased in response to adiponectin treatment in vivo (Figure 4E). In addition to BAT, UCP1 levels in cold-exposed sWAT and eWAT were also significantly downregulated by adiponectin (Figure 4E). We validated these findings using immunohistochemical staining, showing that UCP1-positive signals were sharply decreased in adiponectin-treated cold-exposed iBAT, sWAT, and eWAT (Figure 4F). These findings demonstrate that cold-induced suppression of adiponectin is, at least in large part, responsible for cold-induced atherosclerotic plaque development and instability in the ApoE^−/−^ mouse model.

### Clinical Relevance

To translate our findings to clinical relevance, we conducted a pilot experiment by recruiting human subjects to our studies. We should emphasize that it was notoriously difficult to conduct the cold-exposure experiment in human subjects, and the majority of requested individuals were unwilling to collaborate on this project. We therefore could perform a pilot experiment by recruiting a small number of individuals. Initially, we recruited 14 individuals; their information and disease history are shown in Table S1. Individuals who had a history of smoking, drinking, disease, or medication experiences within the last 2 months were excluded. After blood lipid analysis, we finally recruited five individuals who had high levels of plasma LDL. After 2-day (2 hr/day) exposure to 16°C with standard seminude cloth, plasma levels of LDL cholesterol were measured. Consistent with mouse models, plasma levels of LDL cholesterol were significantly increased as compared with the levels prior to cold exposure (Figure 4G). Importantly, plasma levels of adiponectin were also significantly reduced, as seen in mouse models (Figure 4G). This pilot human study further validates the clinical relevance of our mouse studies. We admit the fact that only a small number of human subjects were recruited at this time, and this pilot study may lack a sufficient statistical power to justify a definite conclusion.

## Discussion

In two genetic atherosclerotic mouse models, we provide compelling evidence that cold acclimation activates lipolysis, leading to high levels of blood cholesterol and LDL cholesterol, especially VLDL and small LDL remnants, which are the core lipids of atherosclerotic plaques. There are two possible mechanisms that potentially underlie the cold-induced hypercholesterolemia: elevated synthesis or defective clearance. Since the same phenotype was also observed in the Ldlr^−/−^, the cold-induced hypercholesterolemia suggests that elevated synthesis might be the underlying mechanism. Indeed, we show that levels of VLDL and small LDL, but not HDL, were markedly increased during cold exposure. Consistent with this notion, LDL synthetic enzymes and transcription factors were also markedly upregulated. However, the causal link between cold-induced BAT or BRITE activation and increased cholesterol synthesis warrants further mechanistic investigation. One of the intriguing findings is that high plasma levels of VLDL and small LDL remnants are only present in ApoE^−/−^ and Ldlr^−/−^ mice, but not in cold-exposed healthy mice. While molecular mechanisms underlying cold-induced differential effects in these genetically manipulated and healthy mice warrant further investigation, this difference may simply reflect the fact that fully functional clearance in healthy mice would be able to reduce hypercholesterolemia to a physiological level despite the high rate of cholesterol synthesis. In ApoE^−/−^ and Ldlr^−/−^ mice, the existence of a defective clearance function of plasma lipoproteins and cholesterols may result in further accumulation of VLDL and small LDL remnants when they are synthesized at high levels. Another possibility is that during cold exposure lipoproteins in ApoE^−/−^ and Ldlr^−/−^ mice undergo substantial remodeling and would no longer be cleared appropriately, leading to plasma accumulation of small LDL remnants.

To link cold-induced lipolysis to atherosclerotic plaque growth, we generated ApoE^−/−^/UCP1^−/−^ mice in which *Apoe* and *Ucp1* genes were completely deleted. As expected, deletion of UCP1 virtually completely protected animals from cold-induced atherosclerotic plaque growth. Although the detailed molecular link between UCP1 expression and hypercholesterolemia is not known at this time, activation of the sympathetic nervous system is essentially required for the cold-induced hypercholesterolemia and atherosclerotic plaque development. We showed that blocking β3-adrenergic receptor using a specific inhibitor virtually completely prevented cold-induced hypercholesterolemia and accelerated plaque development, demonstrating that cold-induced sympathetic activation is crucial for this pathological process. Thus, the sympathetic activation-UCP1 axis is involved in cold-associated hyperlipidemia that contributes to cold-induced accelerated development of atherosclerotic plaques. An independent line of lipolysis evidence was provided using a known lipolysis inhibitor acipimox, which inhibits atherosclerotic plaque development by decreasing the circulating levels of LDL remnants. It appears that increased lipolysis and cholesterol are two cohesively linked processes because inhibition of cholesterol synthesis by simvastatin results in a similar antiatherosclerotic effect. The detailed molecular links by which lipolysis modulates cholesterol synthesis warrant further mechanistic investigation.

Interestingly, circulating adiponectin levels were significantly decreased in cold-exposed mice, and adiponectin has recently been reported to suppress lipolysis (Qiao et al., 2011). Consistent with our findings, activation of the sympathetic nervous system by cold exposure has been reported to suppress circulating levels of adiponectin (Imai et al., 2006). However, the functional aspects of adiponectin in modulation of blood lipid profiles have not been studied. In the present study, we describe our surprising findings that systemic delivery of adiponectin protein to cold-exposed ApoE^−/−^ mice could virtually completely prevent atherosclerotic plaque growth and instability. In a genetic model, crossbreeding between adiponectin^−/−^ mice with ApoE^−/−^ or Ldlr^−/−^ mice did not produce an obvious phenotype of accelerated atherosclerotic plaque development (Nawrocki et al., 2010). The key difference between this genetic model and our cold-induced model is the activation of UCP1-dependent lipolysis. Perhaps without activation of lipolysis as a driving force of alteration of plasma lipid profiles, deletion of adiponectin alone may be insufficient to study its role in accelerated plaque development and instability. Although genetic deletion of adiponectin may provide important clues to the molecular mechanisms of adipokine-induced physiological changes, the relevance of this genetic model to human patients who do not carry genetic deletion of adiponectin is questionable.

Mechanistically, we showed that administration of adiponectin markedly suppresses UCP1 expression at both mRNA and protein levels, leading to ablation of cold-induced activation of BAT and BRITE. Thus, adiponectin inhibits cold-induced UCP1-dependent lipolysis, which essentially contributes to atherosclerotic development in our mouse models. Interestingly, another study showed that induction of UCP1 expression in the vessel wall also accelerated atherosclerotic plaque development in major arteries of mice (Bernal-Mizrachi et al., 2005). This previous study showed that local expression of UCP1 in the vessel wall plays an important role in atherosclerotic plaque development. In the present study, we show that cold-induced adipose UCP1 expression is essential for atherosclerotic plaque development. Nevertheless, this previous study and our present findings point to the role of UCP1 in plaque development. Perhaps upregulation of UCP1, independent of cell types and location, could trigger expression of other factors that subsequently regulate the process of lipolysis. This possibility warrants future investigation.

For healthy human individuals without preatherosclerotic lesions, cold-induced BAT activity is probably beneficial for health improvement. In supporting this hypothesis, cold exposure of genetically nonmanipulated healthy mice did not increase plasma LDL cholesterol levels due to upregulation of Ldlr and ApoE, both involved in high rates of cholesterol clearance. The same is probably true for healthy humans. Patients with preatherosclerotic lesions may have to pay a “high price” for their health when exposed to cold or living in a cold climate. In this group of patients, protection from cold may significantly prevent development of atherosclerosis-associated MI and stroke. The similar lipolysis-mediated CVD mechanism may also apply to other situations such as the drug-, chronic disease-, or even physical exercise-induced lipolysis. Given the high incidence of CVD as the leading cause of human mortality, a rigorous clinical trial should be designed in the future to study the association of the cold-induced BAT activity and development of CVD. Such a clinical study will provide invaluable information for the development of approaches for the prevention and treatment of CVD and other metabolic disorders.

## Experimental Procedures

### Animal and Human Subjects

All animal and human studies complied with the Management Rules of the Chinese Ministry of Health and were approved by the Ethical Committee of Shandong University Qilu Hospital (See the Supplemental Information for more information).

### Generation of UCP1^−/−^ and ApoE^−/−^ Double Knockout Mice

For generation of UCP1^−/−^ and ApoE^−/−^ double knockout mice, UCP1^−/−^ and ApoE^−/−^ were crossbred, and the double knockout strain was identified by genotype using the tail DNA and a PCR-based method. The specific primers for PCR reaction were provided by the commercial companies that supplied these genetic mouse strains (Jackson Laboratory and the Pennington Biomedical Research Center).

### Treatment with Simvastatin, Acipimox, β3-Adrenergic Antagonist SR 59230A, or Adiponectin

Shortly before cold or thermoneutral temperature exposure, 8-week-old ApoE^−/−^ mice (n = 15/group) were treated with or without simvastatin (50 mg/kg/d, Tocris Bioscience), a lipolysis inhibitor acipimox (added into drinking water; 0.05% w/w, Sigma-Aldrich), a selective β3-adrenergic antagonist SR 59230A hydrochloride (i.p. 5 mg/kg/day, Tocris Bioscience), or a recombinant adiponectin (i.p. 1.5 mg/kg/day Prospec). At the end of the 4-week treatment, mice were sacrificed, and blood and tissue samples were collected for various analyses.

### Immunohistochemistry

Tissues prepared from cryosections or paraffin-embedded samples (5 μm thickness) were immunohistologically stained with various primary antibodies, including a nonimmune immunoglobulin G (IgG) (see above), followed by further staining with secondary antibodies as previously described (Zhang et al., 2010, 2011). For staining of blood vessels in the adipose tissue, whole-mount technology was used, and positive signals were detected using a confocal microscope (Zeiss 710). See the Supplemental Information for more information.

### Statistical Analysis

The data were shown as mean determinants ± SEM. Variables with skewed distribution were analyzed by the Mann-Whitney test. For normal distribution data, differences between two groups were assessed by unpaired Student’s t test, and comparison of multiple groups involved the use of ANOVA.

## Accession Numbers

The Gene Expression Omnibus (GEO) accession number for the data set reported in this paper is GSE13432.

## Supplemental Information

Document S1. Supplemental Experimental Procedures, Figures S1–S4, and Table S1
