# Supplementary material for: Cold Exposure Promotes Atherosclerotic Plaque Growth and Instability via UCP1-Dependent Lipolysis
Source: Cell Metab. 2013 Jul 2;18(1):118–29. doi: 10.1016/j.cmet.2013.06.003 (PMC3701322; doi:10.1016/j.cmet.2013.06.003)
Supplement: Document S1. Supplemental Experimental Procedures, Figures S1–S4, and Table S1 [file mmc1.pdf]

Cell Metabolism, Volume *18*

Supplemental Information

Cold Exposure Promotes

Atherosclerotic Plaque Growth and Instability

via UCP1-Dependent Lipolysis

Mei Dong, Xiaoyan Yang, Sharon Lim, Ziquan Cao, Jennifer Honek, Huixia Lu, Cheng Zhang, Takahiro Seki, Kayoko Hosaka, Eric Wahlberg, Jianmin Yang, Lei Zhang, Toste Länne, Baocun Sun, Xuri Li, Yizhi Liu, Yun Zhang, and Yihai Cao

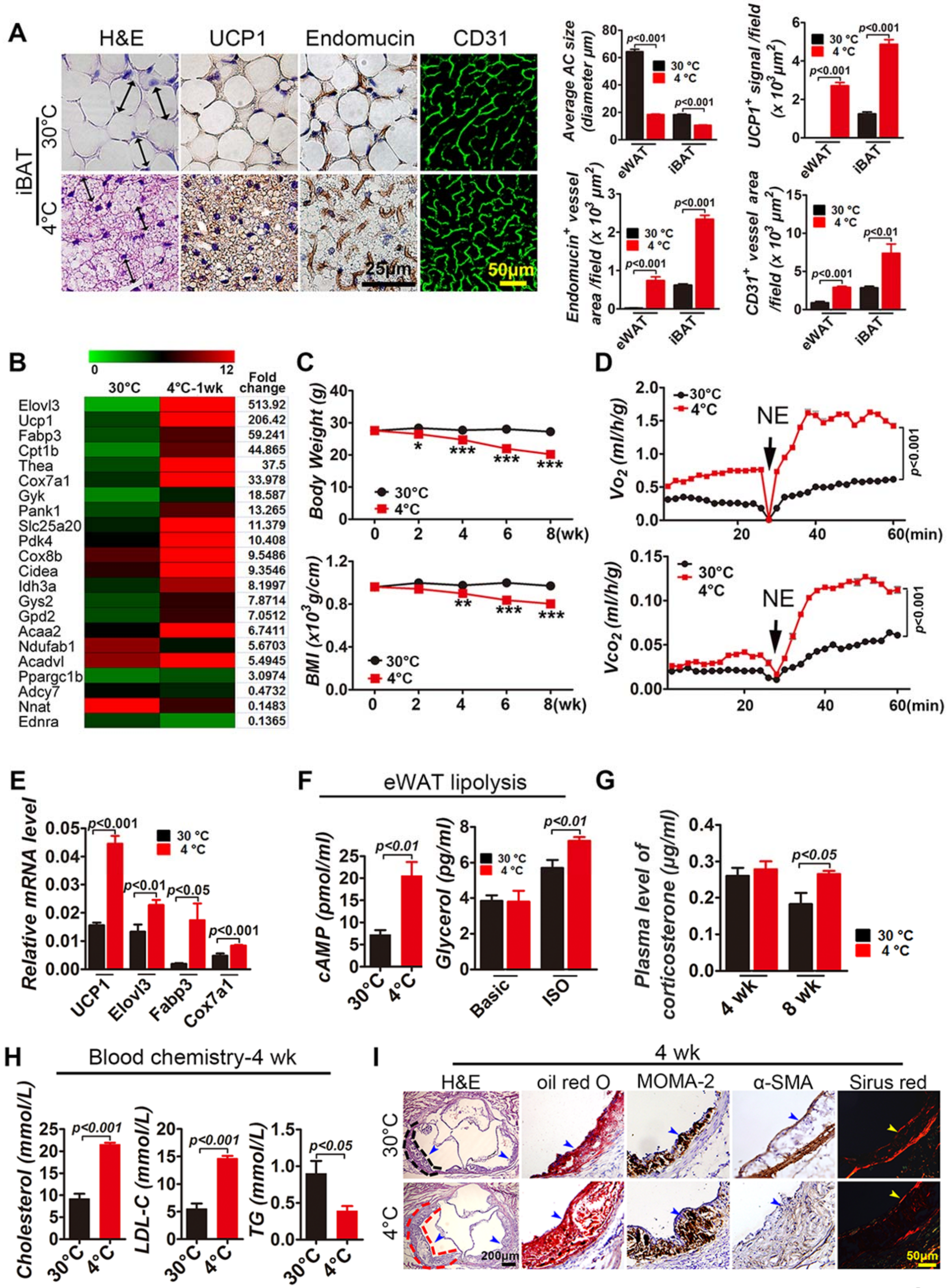
A
H&E
UCP1
Endomucin
CD31
iBAT
30°C
4°C
25μm
50μm
Average AC size (diameter μm)
UCP1+ signal /field (x 10^3 μm^2)
Endomucin+ vessel area /field (x 10^3 μm^2)
CD31+ vessel area /field (x 10^3 μm^2)
30 °C
4 °C
p<0.001
p<0.01
eWAT
iBAT
B
0
12
30°C
4°C-1wk
Fold change
Elovl3 513.92
Ucp1 206.42
Fabp3 59.241
Cpt1b 44.865
Thea 37.5
Cox7a1 33.978
Gyk 18.587
Pank1 13.265
Slc25a20 11.379
Pdk4 10.408
Cox8b 9.5486
Cidea 9.3546
Idh3a 8.1997
Gys2 7.8714
Gpd2 7.0512
Acaa2 6.7411
Ndufab1 5.6703
Acadvl 5.4945
Ppargc1b 3.0974
Adcy7 0.4732
Nnat 0.1483
Ednra 0.1365
C
Body Weight (g)
BMI (x10^3 g/cm)
8(wk)
D
Vo2 (ml/h/g)
Vco2 (ml/h/g)
NE
60(min)
p<0.001
E
Relative mRNA level
p<0.001
p<0.01
p<0.05
UCP1
Elovl3
Fabp3
Cox7a1
F
eWAT lipolysis
cAMP (pmol/ml)
Glycerol (pg/ml)
p<0.01
Basic
ISO
G
Plasma level of corticosterone (μg/ml)
p<0.05
4 wk
8 wk
H
Blood chemistry-4 wk
Cholesterol (mmol/L)
LDL-C (mmol/L)
TG (mmol/L)
p<0.001
p<0.05
I
4 wk
H&E
oil red O
MOMA-2
α-SMA
Sirus red
200μm
50μm

Figure S1

Figure S1. Cold-Induced WAT-BRITE Conversion, BAT Activation, Blood Chemistry, and Atherosclerotic Plaque in 4-Week-Old ApoE$^{-/-}$ Mice, Related to Figure 1

(A) Tissue sections of iBAT exposed to 4°C or 30°C for 4 wk were stained with H&E, mitochondrial UCP1, endomucin, and CD31. Double-arrowed bars indicate diameters in some adipocytes. Average diameters of adipocytes of 4-wk-4°C- or 4-wk-30°C-eWAT and -iBAT (10 random fields/group x 5 mice). Quantification of UCP1^{+} signals, endomucin^{+} and CD31^{+} microvessel density of 4-wk-4°C- or 4-wk-30°C-eWAT and -iBAT (10 random fields/group x 5 mice). (B) Expression profiles of lipolysis-related genes in 1-wk-4°C- and 1-wk-30°C- iWAT (3 repeats/group). (C) Body weight and BMI of pair-fed 4°C- and 30°C-ApoE-/- mice (n=20/group). *$p<0.05$; ***$p<0.001$. (D) Metabolic rate of O_2 consumption and CO_2 production in response to NE of pair-fed 4°C- and 30°C-ApoE-/- mice (n=7/group). (E) qPCR analysis of expression levels of lipolysis-related genes in 4-wk-4°C- and 4-wk-30°C-iWAT of ApoE-/- mice (n=6/group). (F) Measurement of cAMP and glycerol levels in 4-wk-4°C- and 4-wk-30°C-eWAT (n=5/group). (G) Measurement of plasma levels of corticosterone in 4°C- and 30°C-exposed groups of ApoE-/- mice (n=10/group). (H) Blood chemistry analysis of TG, cholesterol, LDL-cholesterol and glycerol (n=10/group). (I) Histological analysis of 30°C-, 4°C-aorta roots stained with H&E, Oil Red O, MOMA-2, α-SMA, or Sirus Red. (n=10/group).

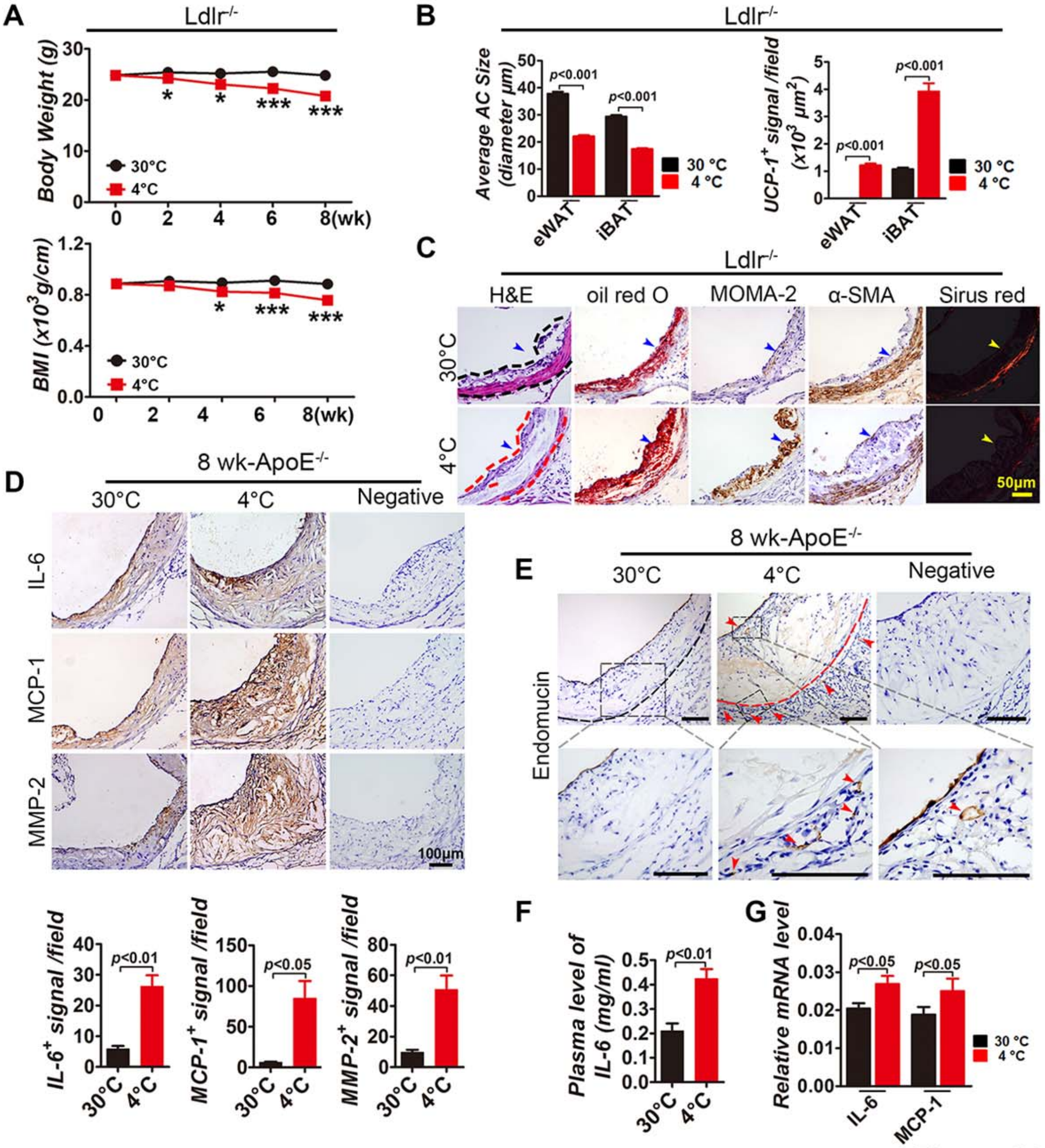

A
Ldlr-/-
Body Weight (g)
30°C
4°C
0
2
4
6
8(wk)
BMI (x10^3g/cm)
B
Ldlr-/-
Average AC Size (diameter μm)
p<0.001
eWAT
iBAT
30 °C
4 °C
UCP-1+ signal /field (x10^3 μm^2)
C
Ldlr-/-
H&E
oil red O
MOMA-2
α-SMA
Sirus red
30°C
4°C
50μm
D
8 wk-ApoE-/-
30°C
4°C
Negative
IL-6
MCP-1
MMP-2
100μm
IL-6+ signal /field
p<0.01
MCP-1+ signal /field
p<0.05
MMP-2+ signal /field
E
8 wk-ApoE-/-
30°C
4°C
Negative
Endomucin
F
Plasma level of IL-6 (mg/ml)
p<0.01
G
Relative mRNA level
p<0.05
IL-6
MCP-1
30 °C
4 °C

Figure S2

Figure S2. Cold-Induced Body Weight, BMI, Adipocyte, and Atherosclerotic Plaque in Ldlr$^{-/-}$ Mice; Inflammation-Related Cytokines and Angiogenesis in Cold-Exposed 8-Week-Old ApoE$^{-/-}$ Mice, Related to Figure 2

(A) Body weight and BMI of pair-fed 4°C- and 30°C-Ldlr$^{-/-}$ mice (n=20/group). *p<0.05; ***p<0.001. (B) Average diameters of adipocytes and quantification of UCP1^{+} signals of 8-wk-4°C- or 8-wk-30°C-eWAT and -iBAT (10 random fields/group x 5 mice). (C) Histological analysis of 8-wk-4°C- and 8-wk-30°C-aorta roots by staining with H&E, Oil Red O, MOMA-2, α-SMA, or Sirus Red. Dashed lines encircle some parts of atherosclerotic plaques and arrowheads in different panels point to positive signals (n=8/group). (D) Histological analysis and its quantification of 8-wk-4°C- and 8-wk-30°C-aorta roots by staining with IL-6, MCP-1 and MMP-2 (n=10/group). Non-immune IgG was used as negative controls. (E) Endomucin+ microvessels were present within the atherosclerotic plaques and in the adventitial region of 8-wk-4°C-atherosclerotic plaques. Non-immune IgG was used as negative controls. Dashed lines encircle some parts of atherosclerotic plaques and arrowheads in different panels point to positive signals (n=10/group). Scale bars in all panels are 100 μm. (F) Expression level of circulating IL-6 of 8-wk-4°C- and 8-wk-30°C-ApoE-/- mice (n=10/group). (G) qPCR analysis of expression levels of IL-6 and MCP-1 in 8-wk-4°C- and 8-wk-30°C-iWAT (n=6/group).

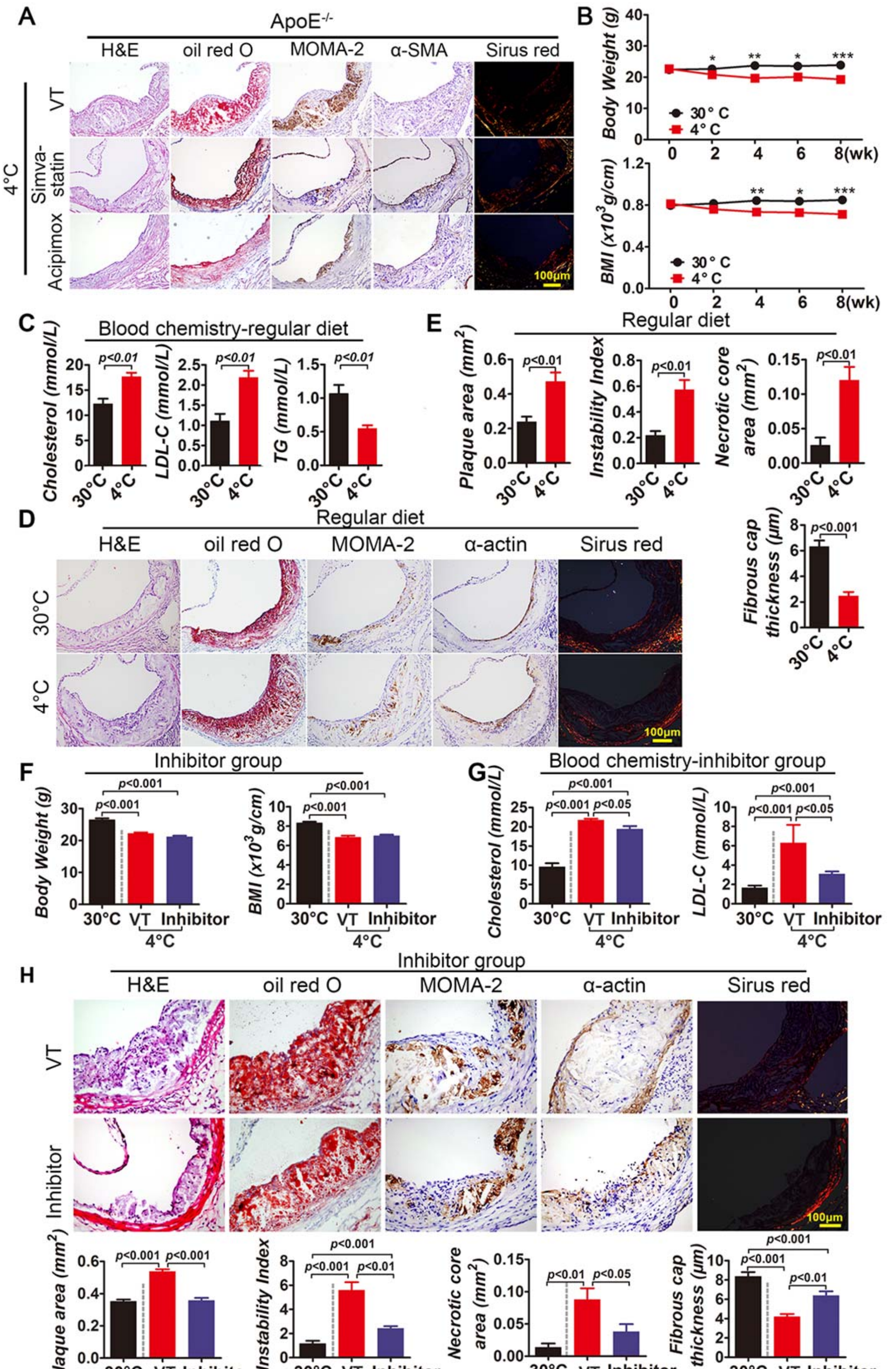

A
ApoE-/-
H&E
oil red O
MOMA-2
α-SMA
Sirus red
4°C
VT
Simva-statin
Acipimox
100μm
B
Body Weight (g)
30° C
4° C
0
2
4
6
8(wk)
BMI (x10³g/cm)
C
Blood chemistry-regular diet
Cholesterol (mmol/L)
LDL-C (mmol/L)
TG (mmol/L)
p<0.01
30°C
4°C
D
Regular diet
H&E
oil red O
MOMA-2
α-actin
Sirus red
30°C
4°C
100μm
E
Regular diet
Plaque area (mm²)
Instability Index
Necrotic core area (mm²)
Fibrous cap thickness (μm)
p<0.01
p<0.001
F
Inhibitor group
Body Weight (g)
BMI (x10³g/cm)
p<0.001
30°C
VT
Inhibitor
4°C
G
Blood chemistry-inhibitor group
Cholesterol (mmol/L)
LDL-C (mmol/L)
p<0.001
p<0.05
H
Inhibitor group
H&E
oil red O
MOMA-2
α-actin
Sirus red
VT
Inhibitor
100μm
Plaque area (mm²)
Instability Index
Necrotic core area (mm²)
Fibrous cap thickness (μm)
p<0.001
p<0.01
p<0.05
30°C
VT
Inhibitor
4°C

Figure S3

Figure S3. Antiatherosclerotic Effects by Simvastatin and Acipimox; Cold-Induced Alteration of Body Weight, BMI, Blood Chemistry, and Athersclerotic Plaques in Regular Diet-Fed ApoE$^{-/-}$ Mice and SR-59230A-Treated ApoE$^{-/-}$ Mice, Related to Figure 3

(A) Histological analysis of vehicle-, simvastatin- and acipimox-treated groups stained with H&E, Oil Red O, MOMA-2, α-SMA, or Sirus Red. (n=10/group). (B) Body weight and BMI of 8 wk-30°C-, 8 wk-4°C-pair-fed-ApoE$^{-/-}$mice with regular diet (n=10/group). (C) Blodd chemistry of 30°C-, 4°C-exposed groups with regular diet (n=10/group). (D) Histological analysis of 30°C-, 4°C-aorta roots stained with H&E, Oil Red O, MOMA-2, α-SMA, or Sirus Red. (n=10/group). (E) Quantification of plaque areas, instability index, necrotic core area and fibrous cap thickness of 30°C-, 4°C-exposed groups (n=10/group). (F) Body weight and BMI of pair-fed 4 wk-4°C-control- and 4 wk-4°C- SR-59230A-treated ApoE$^{-/-}$mice (n=10/group). (G) Blood chemistry of vehicle- and SR-59230A-treated groups (n=10/group). (H) Histological analysis of vehicle- and SR-59230A-treated groups stained with H&E, Oil Red O, MOMA-2, α-SMA, or Sirus Red. (n=10/group). Quantification of plaque areas, instability index, necrotic core area and fibrous cap thickness of vehicle- and SR-59230A-treated groups (n=10/group). VT=vehicle-treated; inhibitor=SR-59230A-treated.

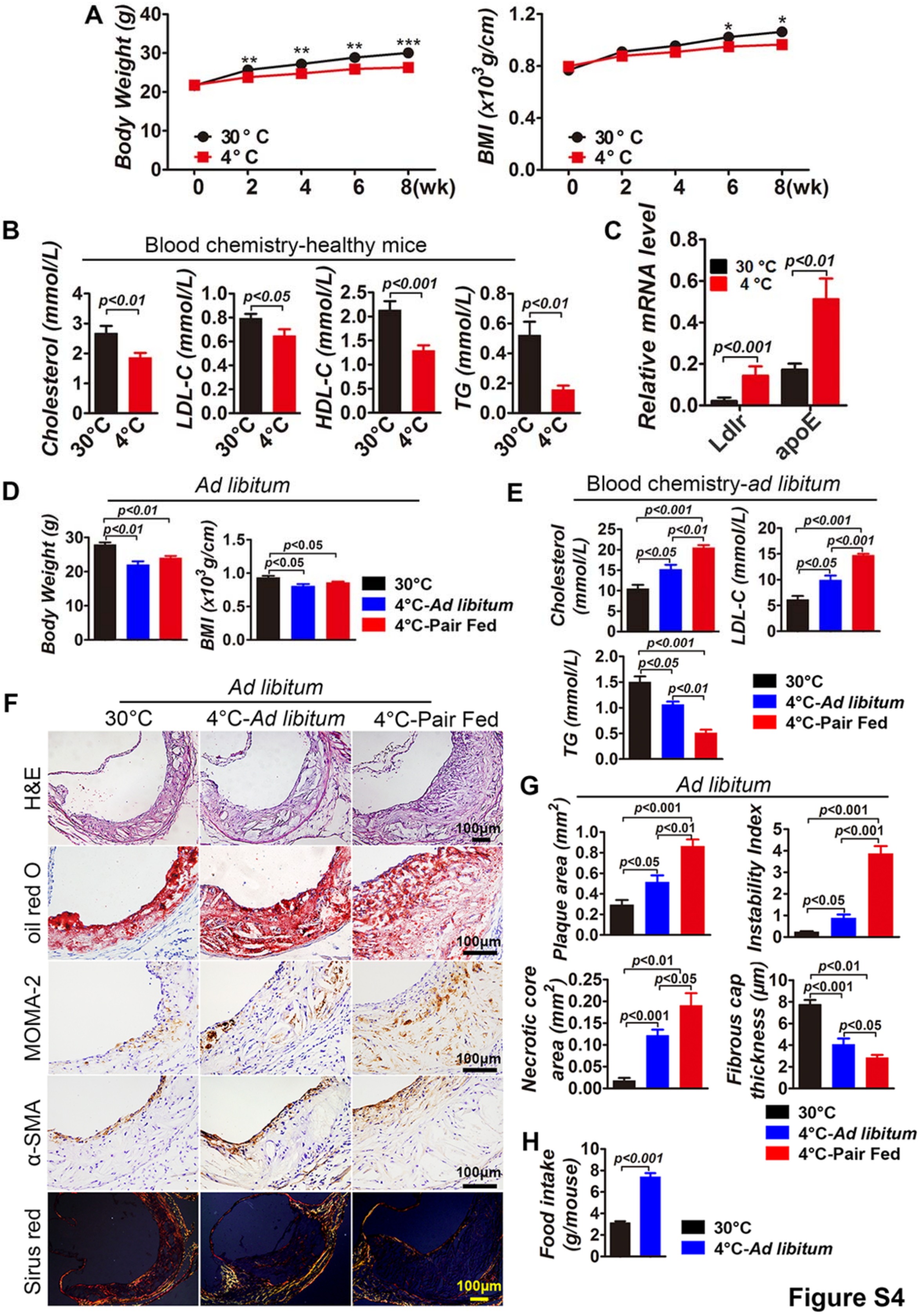

A
Body Weight (g)
30° C
4° C
8(wk)
BMI (x10^3 g/cm)
B
Blood chemistry-healthy mice
Cholesterol (mmol/L)
LDL-C (mmol/L)
HDL-C (mmol/L)
TG (mmol/L)
p<0.01
p<0.05
p<0.001
C
Relative mRNA level
30 °C
4 °C
Ldlr
apoE
D
Ad libitum
E
Blood chemistry-ad libitum
30°C
4°C-Ad libitum
4°C-Pair Fed
F
H&E
oil red O
MOMA-2
α-SMA
Sirus red
100μm
G
Plaque area (mm^2)
Instability Index
Necrotic core area (mm^2)
Fibrous cap thickness (μm)
H
Food intake (g/mouse)

Figure S4

Figure S4. Cold-Induced Alterations of Body Weight, BMI, Blood Chemistry, and Athersclerotic Plaques in Wild-Type Mice and Ad Libitum Fed Mice, Related to Figure 4

(A) Body weight and BMI of 8 wk-30°C-, 8 wk-4°C-pair-fed-wt mice (n=10/group). (B) Blood chemistry of 8 wk-30°C-, 8 wk-4°C-pair-fed-wt mice (n=10/group). (C) Hepatic mRNA levels of Ldlr and ApoE of 8 wk-30°C-, 8 wk-4°C-pair-fed-wt mice (n=10/group).

(D) Body weight and BMI of 8 wk-30°C-, 8 wk-4°C-pair-fed- and 8 wk-4°C-*ad-libitum*-fed $ApoE^{-/-}$mice (n=10/group). (E) Blood chemistry of 30°C-, 4°C-pair-fed- and 4°C-*ad-libitum*-fed $ApoE^{-/-}$mice (n=10/group). (F) Histological analysis of 30°C-, 4°C-pair-fed- and 4°C-*ad-libitum*-fed aorta roots stained with H&E, Oil Red O, MOMA-2, α-SMA, or Sirus Red. (n=10/group). (G) Quantification of plaque areas, instability index, necrotic core area and fibrous cap thickenss of 30°C-, 4°C-pair-fed- and 4°C-*ad-libitum*-fed $ApoE^{-/-}$mice (n=10/group). (H) Quantification of daily food intake of 30°C- and 4°C-*ad-libitum*-fed $ApoE^{-/-}$mice (n=10/group).

Table S1. Clinical Characteristics and Blood Chemistry Parameters before Cold Exposure, Related to Figure 4G

Subject No.	Gender	Age (years)	Smoking history	Drinking history	Medication history	Diabetes history	TC (mmol/L)	LDL-C (mmol/L)	HDL-C (mmol/L)	TG (mmol/L)
1	Male	39	Y	N	N	N	3.92	3.01	1.16	1.26
**2 #**	**Male**	**41**	**N**	**N**	**N**	**N**	**5.00**	**3.61**	1.05	1.57
**3 #**	**Male**	**46**	**N**	**N**	**N**	**N**	**6.76**	**4.24**	1.25	1.89
4	Male	43	N	N	N	N	4.21	2.32	1.21	1.35
5	Male	42	N	N	N	N	3.68	1.97	1.46	0.81
6	Male	47	Y	N	N	N	4.86	3.16	1.21	1.11
**7 #**	**Male**	**44**	**N**	**N**	**N**	**N**	**5.32**	**3.79**	1.12	1.47
8	Male	40	N	N	N	N	3.55	1.89	1.42	1.38
9	Male	42	N	N	Y	N	4.11	2.35	1.26	1.17
**10 #**	**Male**	**45**	**N**	**N**	**N**	**N**	**6.61**	**4.56**	1.01	1.77
11	Male	41	N	Y	N	N	3.75	2.05	1.45	1.58
**12 #**	**Male**	**43**	**N**	**N**	**N**	**N**	**5.93**	**4.12**	1.04	1.62
13	Male	40	N	Y	N	N	3.52	1.81	1.57	0.94
14	Male	44	N	N	N	N	4.02	2.13	1.39	1.22

If the volunteer subjects have any smoking ,drinking, medication history (statins) in the past two months, and any diabetic or other disease history, Y is short for Yes, and N stands for No. The volunteer subjects' numbers with # are those individuals who were chosen for this study.

Supplemental Experimental Procedures

Animals and Human Subjects

Male wild type (*wt*) and ApoE$^{-/-}$ mice in C57BL/6 background at the age of 8-wk were purchased from Beijing Wei Tong Li Hua Experimental Animal Technology Co. LTD (Beijing, China). Male Ldlr$^{-/-}$ mice in C57BL/6 background at the age of 8-wk were purchased from the Animal Model Institute, Nanjing University (Nanjing, China). UCP1$^{-/-}$ mice in C57BL/6 background were kindly provided by Pennington Biomedical Research Center (Baton Rouge, LA). Mice were sacrificed by anesthetizing with intraperitoneal (i.p.) injection of 0.8% pentobarbital sodium (60 mg/kg), followed by cervical dislocation. All animal and human studies were complied with the Management Rules of the Chinese Ministry of Health and approved by the Ethical Committee of Shandong University Qilu Hospital.

Reagents

Antibodies used in this study include: A rat anti-mouse PECAM-1 (CD31) monoclonal antibody (BD Pharmingen, San Diego); a rat anti-mouse monoclonal [MOMA-2] monocyte/macrophage antibody (AbD Serotec, Oxford, UK); a rabbit anti-mouse α-SMA antibody (Abcam, Cambridge, UK); a rabbit anti-mouse UCP1 antibody (Abcam, Cambridge, UK); a rabbit anti-mouse endomucin antibody (eBioscience, San Diego) and an Alexa-488 green-labeled goat anti-rat IgG (Molecular Probes, CA). An instant Streptavidin-Peroxidase immunohistochemical kit (Zhongshan Goldenbrige Biotechnology Co.LTD, Beijing, China) was used for HRP staining.

Cold Acclimation

Eight-wk-old male *wt*, $ApoE^{-/-}$, $Ldlr^{-/-}$, or $UCP1^{-/-}$ and $ApoE^{-/-}$ double knockout C57BL/6 mice caged in 22°C were fed with a high-fat-diet (0.25% cholesterol and 15% cocoa butter), (Beijing Wei Tong Li Hua Experimental Animal Technology Co. LTD, Beijing, China) for 4 wks. Each of the strain of mice was randomly divided into two groups (n=20/group) and switched to pair-fed or *ad libitum* feeding with regular diet. In some experimental settings, mice were fed with regular chow throughout the entire experiments. While one group of mice was exposed to 30°C for 4 or 8 wks, the other group were first adapted at 18°C for 1 week, followed by exposure to 4°C for another 3 or 7 wks. The 4°C-cold room was equipped with a ventilation system that allowed cold air to circulate in the room.

Tissue Sample Collection

For collection of blood samples, animals exposed under different conditions were fasted overnight and anesthetized with 0.8% pentobarbital sodium (i.p, 60 mg/kg). Blood was intracardially collected using a syringe. After sacrificing animals, various adipose depots, heart, arteries and liver were dissected. For analysis of mRNA expression, tissue samples were immediately placed in liquid nitrogen. The samples were kept in -80°C until use. For histological analysis, samples were fixed with 4% paraformaldehyde overnight, followed by washing with 1x PBS. A portion of the samples was paraffin-embedded and the rest was used for cryosections.

Blood Lipid Analysis

After collection of blood samples, plasma from each mouse was prepared and stored at -80°C until use. Free fatty acids were measured using a commercially available ELISA kit (Roche, Shanghai, China). Total plasma levels of cholesterol, TG, LDL-cholesterol, and high-density

lipoprotein cholesterol (HDL-C) were measured using commercially available agents (Zhejiang Dongou Biotechnology, Wenzhou, China).

FPLC chromatography was used for detailed analysis of various cholesterol components and TG (n=6/group). Briefly, a aliquot of 100 μl of plasma was chromatographed on a Superose 6 HR10/300 GL column (GE Healthcare, Sweden) and eluted with 1x PBS (PH 7.2) at a speed of 0.5 ml/min. A total number of 60 fractions (0.5 ml/fraction) were collected, and each fraction was analyzed for cholesterol and TG concentrations using standard ELISA Kits (Cayman, Florida). Values from fractions 5-10 were used for calculation of VLDL/IDL, whereas fractions 11-19 and fractions 20-30 represent LDL and HDL respectively.

In Vivo Cholesterol Synthesis

Various groups of $ApoE^{-/-}$ mice (n=6/group) were injected (i.p.) with 20 mCi of $[^3H]$water (PerkinElmer, USA) in 0.1 ml of phosphate saline. One houe after injection, mice were anesthetized and blood samples were immediately collected and used for measurement of plasma $[^3H]$. Liver tissues were homogenized and 200–300 mg of homogenate portions were saponified, and the digitoninprecipitable sterols were isolated as previously described (Dietschy and Spady, 1984). The rates of hepatic cholesterol synthesis were calculated as the mmol of $[^3H]$water incorporated into digitonin-precipitable sterols per hour per gram of tissues.

Measurement of cAMP and Glycerol

Tissue homogenates from eWAT exposed to different temperatures were used to measure the levels of cAMP using a commercially available kit (Cayman, Florida). For detection of glycerol, adipocytes from eWAT were isolated using collagenase II digestion (0.25 mg/ml) at

37°C for 1 h. The single cell suspension was cultured at 37°C for 1 h in the presence or absence of isoproterenol in an AIS buffer containing 125 mM NaCl, 5 mM KCl, 1 mM CaCl2, 2.5 mM MgCl2, 1 mM KH2PO4, 2% BSA, 4 mM glucose, and 25 mM Tris (pH 7.4). The released glycerol was measured using a commercial kit (Sigma, Shanghai, China).

Indirect Calorimetry

Oxygen consumption was measured using an open-circuit system (Sable, USA) as previously described (Xue et al., 2009). Prior to measurement, mice were anesthetized by i.p. injection with pentobarbital at the dose of 90 mg/kg. Animals were immediately transferred to a 33°C metabolic chamber, where O_2 consumption and release of CO_2 were uninterruptedly recorded for 30 min. At the end of 30 min, a single subcutaneous injection of NE at the dose of 20 μg/20g-mouse was administrated. O_2 consumption and release of CO_2 were further recorded for successive 90 min.

Histological Staining

Some tissue samples were stained with H&E using a standard protocol. Oil red O (Sigma, Shanghai, China) at the final concentration of 0.5% was used to stain tissue sections (5 μm thickness) for 10 min. The stained samples were washed with 37°C water for a few seconds. The samples were counter-stained with hematoxylin. Picrosirus red (Sigma, Shanghai, China) at the final concentration of 0.5% was used for staining collagens.

Immunohistochemistry

Tissues prepared from cryosections or paraffin-embedded samples (5 □m thickness) were immunohistologically stained with various primary antibodies including a non-immune IgG (see above), followed by further staining with secondary antibodies as previously described

(Zhang et al., 2010; Zhang et al., 2011). For staining of blood vessels in the adipose tissue, the whole-mount technology was used and the positive signals were detected using a confocal microscope (Zeiss-710, Zeiss, Germany). Atherosclerotic plaque instability index was calculated according to the standard formula: (Oil Red O^+ signal plus MOMA-2^+ signal)/ (α-SMA^+ signal plus collagen I^+ signal) (Zhang et al., 2010).

Statistical Analysis

The data were shown as mean determinants ± SEM. Variables with skewed distribution were analyzed by the Mann-Whitney test. For normal distribution data, differences between two groups were assessed by unpaired Student's *t*-test, and comparison of multiple groups involved the use of ANOVA.

SUPPLEMENTAL REFERENCES

Dietschy, J.M., and Spady, D.K. (1984). Measurement of rates of cholesterol synthesis using tritiated water. J. Lipid Res. 25, 1469-1476.

Xue, Y., Petrovic, N., Cao, R., Larsson, O., Lim, S., Chen, S., Feldmann, H.M., Liang, Z., Zhu, Z., Nedergaard, J., et al. (2009). Hypoxia-independent angiogenesis in adipose tissues during cold acclimation. Cell Metab. 9, 99-109.

Zhang, C., Zhao, Y.X., Zhang, Y.H., Zhu, L., Deng, B.P., Zhou, Z.L., Li, S.Y., Lu, X.T., Song, L.L., Lei, X.M., et al. (2010). Angiotensin-converting enzyme 2 attenuates atherosclerotic lesions by targeting vascular cells. Proc. Natl. Acad. Sci. USA 107, 15886-15891.

Zhang, D., Hedlund, E.M., Lim, S., Chen, F., Zhang, Y., Sun, B., and Cao, Y. (2011). Antiangiogenic agents significantly improve survival in tumor-bearing mice by increasing tolerance to chemotherapy-induced toxicity. Proc. Natl. Acad. Sci. USA 108, 4117-4122.
